# What Is Not Conceptualized Is Not Measured: Towards Healthier Societies: Comment on "How to Build Healthy Societies: A Thematic Analysis of Relevant Conceptual Frameworks"

**DOI:** 10.34172/ijhpm.9184

**Published:** 2026-05-23

**Authors:** Erica Di Ruggiero

**Affiliations:** Social and Behavioural Health Sciences Division, Institute of Health Policy, Management & Evaluation, Dalla Lana School of Public Health, University of Toronto, Toronto, ON, Canada.

**Keywords:** Equity, Measurement, Intersectoral Interventions, Power, Governance, Healthier Societies

## Abstract

Global geopolitical conflicts, pandemics, the climate crisis or rising inequities within and between countries alongside other intersecting crises all impede the pursuit of healthier societies. This commentary highlights gaps in how framing and measurement approaches reflect narrower biomedical conceptualizations of health. These gaps also relate to whether and how power is analysed, and how interventions that contribute to the (re)building of healthier societies are designed, implemented and evaluated. Dominant measurement approaches in health that prioritize individual measurement of problems and solutions at the expense of whole-of-society intersectoral interventions, and their governance are among some of the reasons. More holistic and explicit measurement equity and well-being (as opposed of disease) are required. Theory-informed research that interrogates values, social norms and ideologies, power asymmetries and that centres the study of complex context-sensitive policy and program interventions should inform future inquiries.

## Background

 Global, sociopolitical, cultural, and environmental drivers that contribute to health, well-being and to creating healthier societies are the subject of extensive scholarship. In their review article entitled “*How to Build Healthy Societies: A Thematic Analysis of Relevant Conceptual Frameworks,*”^1^ Nambiar and colleagues analysed documents published since Canada’s Lalonde report in 1974 (a report considered at the time as advancing a transformative perspective on health). The authors critically interrogate the public policy levers and “enablers” of healthy societies. They thoughtfully discuss several levers including redefining measures of progress and intersectoral action and enablers such as political will and accountability to build healthier societies. Perhaps not surprisingly, they conclude that many of the documents are more often framed in positivist and reductionist ways, a power analysis is often somewhat wanting, and rights-based approaches are overlooked.^1^ It is also not unexpected that neoliberal and biomedical approaches to health prevail in these frameworks. This trend persists despite numerous calls to shift the emphasis in health upstream and away from a lifestyle drift approach, where societal norms reinforce individual responsibility for adopting healthy behaviours rather than on tackling the structural, political, social, and environmental determinants of health inequities – “the causes of the causes.”^2^

 Dominant measurement approaches predominantly oriented towards the individual measurement of problems can crowd out a focus on measuring the effects of whole-of-society interventions that engage multiple sectors and how they are governed.

 In this commentary, I reflect on gaps in how framing and measurement approaches represent narrower biomedical conceptualizations of health drawing in part on insights from Nambiar and colleagues, and other research examples. These gaps also relate to whether and how power is analysed, and how interventions that contribute to the (re)building of healthier societies are designed, implemented and evaluated. I conclude with some reflections on the implications for future research.

## Framing of Societal Problems

 First coined by the French sociologist, philosopher and complex theorist Edgar Morin in 1993 and further discussed with co-author Anne Brigitte Kern in their 1999 book *Homeland Earth: A Manifesto for a New Millennium,*^3^ the concept of polycrisis has regained attention in recent years. It refers to the accumulation of interconnected and multi-faceted crises that contemporary societies are contending with – be it the COVID-19 pandemic, global conflicts, the climate crisis or rising inequities within and between countries alongside other crises all impede the pursuit of healthier societies. Despite such possible framings through which to understand problems, many health and social problems such as poverty and food insecurity continue to run the risk of being represented by biomedical framings. Biomedicalization is enabled by biopolitical governance that manages populations by combining racialized distinctions with neoliberal principles of market logic and individual responsibility.^4^

 State and non-state actors uphold these logics, and simplistic framings of societal problems and give greater priority to individual-level interventions. How you conceptualize a problem, or a solution also impacts how you measure it. The polycrisis necessitates intersectoral and interdisciplinary program and policy solutions that no one actor or sector can solve alone. In a study we conducted about the contextual factors influencing the uptake of the Sustainable Development Goals (SDGs) by federal policy leaders, one of the key informants observed; *“What Covid has brought us is this understanding that we have to look at things differently and that we need to put building back better at the centre and look for new ways to build back better so…there is an appetite for quality-of-life type of frameworks, SDG frameworks, things that are multifaceted, that are not just drilling down to one number.”*^5^

 Embracing complexity in problem framing, while increasingly recognized as important, requires an understanding of the interconnectedness between elements in a system; however, it also presents conceptual and methodological difficulties for measurement.

## Measurement of Problems and Solutions

 Dominant measurement approaches in health that unduly focus on individualized measurement of problems and solutions rather than the study of whole-of-society intersectoral interventions, and their governance are among some of the reasons.^1^ New approaches to indicators development are needed that explicitly and holistically measure equity and well-being (rather than the absence of a disease), as well as co-benefits, co-harms, and the distribution of co-impacts in the case of whole-of-society intersectoral initiatives. The realization of synergies between co-benefits is of particular relevance to achieving progress towards Agenda 2030. For example, what are the co-benefits and co-harms of sustainable urban transportation for people who do not drive, experience mobility issues, are low-income and/or have longer commutes to employment opportunities or less flexible work schedules? Indicators need to be more contextually sensitive to measuring the harms, benefits and costs for different populations, actors, and/or sectors involved. There are many persisting omissions and silences in what we measure; and, these are particularly consequential for the equity-denied and historically marginalized populations whose voices are not considered in defining what constitutes success in measurement approaches to health and social problems and their intersectoral solutions.

## Measurement of Intersectoral Solutions and Their Governance

 There is increasingly recognition of the need to shift towards intersectoral solutions, but sustainable uptake at scale continues to be a challenge. For example, in an effort to shift the gaze away from individual level measurement towards monitoring intersectoral actions (ie, governance, policy, or programmatic interventions) taken by governments and other societal actors to improve the social determinants of health (SDH) and health equity, an interdisciplinary working group was established to guide a global monitoring system for implementing the 2012 Rio Political Declaration on Social Determinants of Health. The Declaration outlines five action areas including to monitor progress and increase accountability and further reorient the health sector towards promoting health and reducing health inequities.^6^ The working group reviewed the available evidence and selected 36 high-quality SDH action indicators for each of the five action areas. In this analysis, the group found that most indicators were about measuring the SDH (problem) and not the actions (eg, programmatic, governance, or policy). The group noted that the explicit measurement of equity in relation to specific actions was limited, and the measurement of actions related to intersectoral governance and participation were only starting to emerge.^6^

 In 2015 and shortly after the release of the 17 United Nations SDGs signaling the commitment of all 193 member countries to collectively end poverty, protect the planet and ensure that all people enjoy peace and prosperity by 2030, scholars argued for a paradigm shift to make significant progress towards health. To get there, several structural barriers require urgent attention including “to [ensure] leadership for intersectoral coherence and coordination on the structural (including social, economic, political and legal) drivers of health.”^7^ However, insufficient focus is placed on assessing how organizational leadership and governance contribute to health and well-being. “*What COVID has further underscored is that it’s not enough to have one-off projects that link sectors. What we need is to think about fundamental shifts in the way in which we structure governance at higher levels, we need to understand how we are structuring our budgets and our processes around a social determinants type of perspective*.” This illustrative quote drawn from research conducted with federal policy leaders in Canada underscores the importance of measuring governance processes.^5^ Research efforts need to be directed toward measuring the role of multilevel governance processes including how different institutional logics interact in the design and implementation of intersectoral interventions.

## Power Analysis

 Another critical gap is the limited analysis of power^1,8,9^ The inequitable distribution of power is consequential for marginalized populations because power shapes who controls economic and social resources, whose knowledge counts in research, policy decision-making and influences social norms, policies and institutions. Power is often undertheorized or poorly conceptualized. A narrative review of intersectoral action in the global public health and political science literatures with relevance to the SDGs found that power was undertheorized. While empirical studies analyzed showcase “implementation tips,’ they did not sufficiently explore sociopolitical contextual factors nor did they adequately account for power relations, power dynamics, and power asymmetries in cross-sectoral collaboration.^10^ A relevant exception highlighted in this article is Friel and colleagues’ cross-case study of power relations in intersectoral public policy-making, which point to the compounding influences of racism, sexism, neoliberalism, and biomedicalism on the construction of rules and mandates, which can limit policy-making and have harmful effects for health equity.^11^

 Do governments use their executive power to redistribute goods to promote social and health equity at the expense of other policy priorities or use power to maintain status quo? Elite population subgroups exercise disproportionate power and influence, and their impact must be recognized.^1^ The discussion does need to also extend to the unintended consequences for many populations left behind, and frameworks must be attuned to these differential impacts. It is fundamentally a matter of epistemic injustice in which structurally marginalized population groups continue to be systemically excluded from accessing and making decisions over the resources that impact their health and well-being. Although calls for decolonizing global public health knowledge are drawing attention, these efforts also run the risk of elite capture by Global North institutions and Western derived conceptualizations of health and well-being, and to becoming depoliticized and decontextualized.^12,13^ The creation of healthier societies is after all a deeply contextual and sociopolitical endeavour, requiring an ongoing interrogation of powers and politics.

## Towards Context-Sensitive Policy and Program Interventions

 The contextual mechanisms, which facilitate or disrupt the socio-structural, economic, and environmental conditions in which policy and program interventions are designed and implemented and brought to scale require more attention in research as do the conceptual frameworks used to guide implementation. A scoping review of public health interventions found that context was either ignored, not always well-described, or “controlled for.”^14^ Theory-informed methodological approaches (eg, case studies) that generate rich representations of different features of context – social, cultural, historical, political, and so on are needed to further our understanding of intervention mechanisms and pathways to health, well-being and/or equity. Historical contextual influences (eg, colonialism) that have (re)produced inequities continue to have contemporary influences. Context can help explain why an intervention fails or succeeds to produce a desired change. The more complex the intervention the more likely it is entangled within a multilayered context. Theories and frameworks that explicitly attend to context and that discourage the conceptualization of context, the intervention(s) and implementation processes as separate elements are critical. For example, complexity theory adds value to understanding the role of context as a complex system and characterizing the feedback loops between interventions across sectors.^15^

 In closing, theory-informed research that interrogates values, social norms and ideologies, power asymmetries and that centres the study of complex context-sensitive intersectoral interventions should inform future inquiries.^1,6^ Returning to the polycrisis concept could be instructive in understanding different causal pathways that (re)produce and reinforce crises but also for promoting pathways for understanding the genesis and durability of health- and equity-promoting complex intersectoral interventions. As global public health scholars, we must resist the temptation to continually select and measure the effects of simple and reductionist interventions if we want to advance new knowledge towards healthier, more equitable and sustainable societies.

## Disclosure of artificial intelligence (AI) use

 Not applicable.

## Ethical issues

 Not applicable.

## Conflicts of interest

 Author declares that she has no conflicts of interest.
